# Pathogeny of Gastric Ulcer

**Published:** 1887-08

**Authors:** 


### *PATHOGENY OF GASTRIC ULCER.*

The precise pathogeny of chronic ulcer of the stomach is one of those undetermined questions which have led to considerable speculation, with comparatively little profit. The position, form and nature of the ulcer have done more than any positive demonstration of the vascular lesion to favor the current doctrine of its dependence on arterial blocking. But every one knows the difficulties in the acceptance of this view, not the least being the comparative frequency of the disease in the female sex, and the great preponderance of cases where the ulcer is solitary and seated on the posterior surface near the lesser curvature. Dr. Decker, of Würzburg, has the last word on the subject, (*Berl. Klin. Wochenschrift*, No. 21,) and he advances evidence in support of the initial lesion being traumatic, or rather thermal. Thus, he believes that the contact of hot thickened fluids with the gastric mucosa excites hyperæmia, which becomes localized, and may lead to venous statis and hemorrhage in a limited territory, with all the subsequent necrotic changes. He supports his view not only by reference to the clinical history of cases of gastric ulcer, (he points to the great prevalence of gastric ulcer among cooks, who habitually test the flavor of their dishes when very hot,) but by two experiments on dogs into whose stomach food heated to 50° C was introduced. In one of the animals a patch of hyperæmia, with hemorrhage between the gastric mucosa and muscularis near the lower curvature, was found; in the other, a deep ulcer of characteristic shape and position had been produced.—*The Lancet*, May 28, 1887.

